# NRT1.1 Regulates Nitrate Allocation and Cadmium Tolerance in Arabidopsis

**DOI:** 10.3389/fpls.2019.00384

**Published:** 2019-03-27

**Authors:** Shaofen Jian, Jingsong Luo, Qiong Liao, Qiang Liu, Chunyun Guan, Zhenhua Zhang

**Affiliations:** ^1^Southern Regional Collaborative Innovation Centre for Grain and Oil Crops in China, College of Resources and Environmental Sciences, Hunan Agricultural University, Changsha, China; ^2^National Centre of Oilseed Crops Improvement, Hunan Branch, Changsha, China

**Keywords:** *CHL1/NRT1.1/NPF6.3*, *NITRATE REGULATORY GENE 2*, nitrate allocation, cadmium stress, vacuole, Arabidopsis

## Abstract

Abiotic stress induces nitrate (NO_3_^-^) allocation to roots, which increases stress tolerance in plants. NRT1.1 is broadly involved in abiotic stress tolerance in plants, but the relationship between NRT1.1 and NO_3_^-^ allocation under stress conditions is unclear. In this study, we found that Arabidopsis wild-type Col-0 was more cadmium (Cd^2+^)-tolerant than the *nrt1.1* mutant at 20 μM CdCl_2_. Cd^2+^ exposure repressed *NRT1.5* but upregulated *NRT1.8* in roots of Col-0 plants, resulting in increased NO_3_^-^ allocation to roots and higher [NO_3_^-^] root-to-shoot (R:S) ratios. Interestingly, *NITRATE REGULATORY GENE2* (*NRG2*) was upregulated by Cd^2+^ stress in Col-0 but not in *nrt1.1*. Under Cd^2+^ stress, *nrg2* and *nrg2-3chl1-13* mutants exhibited similar phenotypes and NO_3_^-^ allocation patterns as observed in the *nrt1.1* mutant, but overexpression of *NRG2* in Col-0 and *nrt1.1* increased the [NO_3_^-^] R:S ratio and restored Cd^2+^ stress tolerance. Our results indicated that *NRT1.1* and *NRG2* regulated Cd^2+^ stress-induced NO_3_^-^ allocation to roots and that *NRG2* functioned downstream of *NRT1.1*. Cd^2+^ uptake did not differ between Col-0 and *nrt1.1*, but Cd^2+^ allocation to roots was higher in Col-0 than in *nrt1.1*. Stressed Col-0 plants increased Cd^2+^ and NO_3_^-^ allocation to root vacuoles, which reduced their cytosolic allocation and transport to the shoots. Our results suggest that NRT1.1 regulates NO_3_^-^ allocation to roots by coordinating Cd^2+^ accumulation in root vacuoles, which facilitates Cd^2+^ detoxification.

## Introduction

Heavy metal pollution in soil is an important environmental issue worldwide, which gives rise to agricultural and public health concerns (Bertin and Averbeck, 2006; Mohammed et al., 2011; Åkesson et al., 2014). In China, for example, approximately 7% of the soil is cadmium (Cd) contaminated, 0.5% of which is severely polluted (Zhang et al., 2015). Cd can be released to the soil by excessive use of chemical fertilizers and pesticides, utilization of industrial wastewater and sludge, and atmospheric deposition (Woodis et al., 1977; He and Singh, 1994; Wong et al., 2003; Ottosen et al., 2007; Roberts, 2014). Thus, people are being exposed to Cd-associated toxicity via the consumption of cereals and vegetables grown in Cd-contaminated soils (Chaney, 2015). Susceptibility to Cd^2+^ stress is species-specific in plants (Chen, 1996), which provides opportunities to select and breed Cd^2+^-tolerant species/varieties. However, this requires a complete understanding of the underlying mechanisms of Cd^2+^ tolerance in plants.

Nitrate (NO_3_^-^) is one of the two forms of inorganic nitrogen nutrient taken up by terrestrial plants. It also acts as a signal molecule regulating a wide range of genes and biological processes involved in nitrogen utilization, general plant lateral root development, and response to environmental fluctuations (Gowri et al., 1992; Redinbaugh and Campbell, 1993; Gutiérrez et al., 2007; Hirel et al., 2007; Miller et al., 2007; Krouk et al., 2010; Wang et al., 2012; Ruffel et al., 2014; Vidal et al., 2014; Bouguyon et al., 2016). Under normal conditions, most of the absorbed NO_3_^-^ is transported to the shoots for reduction by NR or as a temporary nitrogen pool stored in vacuoles, which is driven by the H^+^ transport energized by the tonoplast H^+^-ATPase and H^+^-PPase (Martinoia et al., 1981, 2007; Han et al., 2016). NO_3_^-^ allocation between the roots and the shoots in plants is important for nitrogen utilization and adaptation to abiotic stresses (Fan et al., 2007; Li et al., 2010; Han et al., 2016). The long-distance transport of NO_3_^-^ is mediated by two nitrate transporters, NRT1.5 and NRT1.8. The former is found in pericycle cells where it is responsible for loading the NO_3_^-^ to the xylem, whereas the latter is found in xylem parenchyma cells, where it contributes to NO_3_^-^ unloading from xylem (Lin et al., 2008; Li et al., 2010; Chen et al., 2012). Under adverse environmental conditions, *NRT1.5* expression is downregulated and *NRT1.8* expression is upregulated in roots. As a result, more NO_3_^-^ allocates to roots, which subsequently increases plant stress tolerance (Li et al., 2010; Chen et al., 2012). This phenomenon, known as SINAR, has been widely observed under several abiotic stresses, including the Cd^2+^ stress (Chen et al., 2012; Zhang et al., 2014). Ethylene and jasmonic acid are involved in the regulation of *NRT1.5* and *NRT1.8* under stress (Zhang et al., 2014).

NRT1.1, which was first cloned in 1993 (Tsay et al., 1993), is essential for NO_3_^-^ uptake and signaling (Ho et al., 2009). In addition to its function as NO_3_^-^ transporter, NRT1.1 also plays important roles in vegetative and reproductive growth (Guo et al., 2001), stomatal opening (Guo et al., 2003), root architecture (Remans et al., 2006; Mounier et al., 2014), and transport of chloride and phytohormones (IAA/ABA/jasmonic acid/GAs) (Tsay et al., 1993; Guo et al., 2002; Krouk, 2016; Corratgé-Faillie and Lacombe, 2017). Moreover, it induces tolerance in plants to abiotic stresses, such as proton stress, salt stress, Cd^2+^ stress, and iron deficiency (Mao et al., 2014; Liu et al., 2015; Abouelsaad et al., 2016; Fang et al., 2016). NRT1.1-mediated plant stress tolerance is closely associated with NO_3_^-^ uptake, assimilation, and accumulation. It has been reported that *NRT1.1* mediates the expression of NO_3_^-^ regulatory genes such as *NRG2* (Ho et al., 2009; Hu et al., 2009; Xu et al., 2016) and NO_3_^-^ assimilation genes such as *NIAs* and *NiR*, as well as some other *NRTs* (Ho et al., 2009; Undurraga et al., 2017). However, the relationship between *NRT1.1* and SINAR, including the control mechanisms, is not well understood.

In this study, we found that *NRT1.1* regulated the expression of *NRT1.5* and *NRT1.8* under Cd^2+^ stress, which increased NO_3_^-^ allocation to roots as a mechanism to resist Cd^2+^ stress. Furthermore, we demonstrated that *NRG2* functioned downstream of *NRT1.1* in regulating NO_3_^-^ allocation. NO_3_^-^ was required to facilitate Cd^2+^ allocation to the roots, where it was mainly stored in the vacuoles for detoxification. Our results provide insights into the effects of the nitrate regulatory gene network on the regulation of plant stress tolerance.

## Materials and Methods

### Plant Materials and Growth Conditions

*Arabidopsis thaliana* wild-type (Col-0), *nrt1.1, nrg2* single and double mutants (*chl1-1, chl1-5, chl1-13, nrg2-1, nrt2-2, nrg2-3chl1-13*), and *NRG2* overexpression lines (*35S::NRG2*/*Col-0, 35S::NRG2*/*chl1-5*) were used in this study. Seeds were sown in nutrition soil and placed in a growth chamber (300 μmol photons⋅m^-2^⋅s^-1^, 16-h photoperiod, 22°C) to germinate and grow. Ten days after sowing, seedlings with two true leaves were transplanted to 600-ml pots and cultivated hydroponically in nutrient medium. The growth medium contained 1.25 mM KNO_3_, 0.625 mM KH_2_PO_4_, 0.5 mM MgSO_4_, 0.5 mM Ca(NO_3_)_2_, 1.25 μM Fe-EDTA, 17.5 μM H_3_BO_3_, 3.5 μM MnCl_2_, 0.25 μM ZnSO_4_, 0.05 μM NaMoO_4_, and 0.125 μM CuSO_4_. The MES buffer (2.5 mM) was used to maintain the pH of the growth medium at pH 5.8. The growth medium was refreshed every 4 days, and the position of the pots was interchanged when refreshing the solution to eliminate any edge effects. Four weeks after sowing, plants were exposed to Cd stress by adding 20 μM CdCl_2_ to the growth medium for 3 days. Control plants were grown without CdCl_2_.

### ^15^N Tracer Assay

Plants of Col-0, *chl1-1, chl1-5* were grown in the normal nutrient medium for 4 weeks, followed by a 12-h treatment with 200 μM CdCl_2_. Then, roots were washed with 0.1 mM CaSO_4_ for 1 min and labeled with 20% atom abundance of Ca(^15^NO_3_)_2_ (pH = 5.8) for 40 min. The roots were washed with 0.1 mM CaSO_4_ and deionized water. Shoots and roots were sampled separately and oven-dried at 70°C for 48 h. Then the samples were pulverized using a TissuLyser (Tissuelyer-48, Jingxin Co. Ltd., China), and ^15^N abundance in samples was measured using a continuous-flow isotope ratio mass spectrometer coupled with a carbon-nitrogen elemental analyzer (ANCA-MS; PDZ Europa).

### Determination of Photosynthetic Parameters

Photosynthesis of fully expanded rosette leaves was measured between 09:00 and 15:00 with a LI-6400 portable photosynthesis system (Li-Cor Inc., Lincoln, NE, United States). The air temperature in the cuvette was 22°C, the PPED was 200 μmol m^-2^ s^-1^, the CO_2_ concentration (*C*_a_) was 500 μmol mol^-1^ (controlled with a CO_2_ mixer), and the VPD was between 1.0 and 1.5 kPa. Before measurement, leaves were placed in the cuvette to adjust for 10 min. Data were recorded after equilibration to a steady state.

Photosynthetic CO_2_-response curves (*A-C*_i_ curves) were measured with a PPFD of 200 μmol m^-2^ s^-1^ at nine points of *C*_a_ (800, 600, 500, 400, 300, 200, 150, 100, 50 μmol mol^-1^). Prior to the measurement, leaves were placed in the cuvette to equilibrate for 30 min under a PPFD of 200 μmol m^-2^ s^-1^ and *C*_a_ of 400 μmol mol^-1^. Temperature and VPD in the cuvette during measurement were maintained as described above. Data were recorded after equilibration to a steady state. *V*_c,max_, *J*_max_, and TPU were calculated according to Sharkey et al. (2007). The parameters *K*_c_, *K*_o_, *R*_d_, and *Γ*^∗^, which were used to calculate *V*_c,max_, *J*_max_, and TPU, were estimated as 27.24 Pa, 16.58 Pa, 1 μmol⋅m^-2^⋅s^-1^, and 3.74 Pa, respectively.

### Determination of Proline, Malondialdehyde (MDA) Concentration, and Superoxide Dismutase (SOD) Activity

Plants were grown in the normal nutrient medium for 4 weeks and then treated with 20 μM CdCl_2_ for 3 days. Fresh leaves were collected to measure proline and MDA concentrations and SOD activity. Proline concentration was measured using the ninhydrin colorimetry method (Bates et al., 1973; Sharma and Dubey, 2005). Briefly, fresh samples (0.5 g) were homogenized in 5 ml of 3% aqueous sulfosalicylic acid using a mortar and a pestle. Homogenates were centrifuged at 10,000 × *g* for 10 min at 4°C, and the supernatants were collected and used for proline analysis. In a test tube, 2 ml of supernatant was added to 2 ml of acidic ninhydrin and 2 ml of glacial acetic acid; then, the mixture was placed in a boiling water bath for 15 min. The processed mixture was extracted with 4 ml toluene by thoroughly vigorous stirring. After keeping the tube at room temperature for 30 min, the absorption of the toluene solution was measured spectrophotometrically at 520 nm. A standard curve was created using L-proline.

Malondialdehyde concentration was measured using the thiobarbituric acid method (Kramer et al., 1991). Fresh samples (0.5 g) were homogenized in 5 ml 5% (w/v) trichloroacetic acid (TCA) with a mortar and a pestle and centrifuged at 10,000 × *g* for 10 min at 4°C. Then, 2 ml supernatant were combined with 2.5 ml TBA reagent [0.6% (w/v) TBA in 10% (w/v) TCA], heated at 100°C for 10 min, cooled, and centrifuged at 4000 × *g* for 10 min. The concentration of MDA was calculated from the absorbance at 600, 532, and 450 nm.

Superoxide dismutase activity was determined according to the method described by Giannopolitis and Ries (1977). The shoot tissues were thoroughly ground with a mortar and a pestle in liquid nitrogen. Then, the samples were homogenized in 0.1 M phosphate buffer containing 0.1 mM EDTA (pH 7.8) and centrifuged at 13,000 × *g* and 4°C for 10 min. The supernatants were used for determining the SOD activity. The SOD reaction mixture contained 50 mM phosphate buffer (pH 7.8), 13 mM methionine, 75 μM nitro blue tetrazolium (NBT), 10 μM EDTA-Na_2_, 2.0 μM riboflavin, and modest volume of extract. Ultrapure water was added to a final volume of 3 ml. The mixtures in the glass test tubes were illuminated for 20 min and the absorbance at 560 nm was measured spectrophotometrically. Identical mixtures that were not illuminated served as blank controls (background).

### Determination of Chlorophyll Concentration

Chlorophyll concentration in rosette leaves was determined by extraction with 80% acetone for 24 h at room temperature in the dark (Wellburn and Lichtenthaler, 1984). The absorbance of the extract was measured at 663 and 645 nm to calculate chlorophyll *a, b*, and total chlorophyll concentrations.

### Measurements of Biomass, Nitrate, and Cd^2+^ Concentration

Four-week-old plants treated with CdCl_2_ for 3 days were sampled and separated into shoots and roots. The samples were oven-dried at 70°C until the weight remained constant (dry weight).

NO_3_^-^ was extracted from the samples using deionized water in a boiling water bath for 15 min and determined spectrophotometrically at 410 nm by nitration of salicylic acid (Cataldo et al., 1975).

Four-week-old plants exposed to CdCl_2_ for 3 days were harvested and washed with 0.1 mM CaCl_2_ for 1 min, followed by rinsing with deionized water for four times. Shoots and roots were separately collected and oven-dried at 70°C until the weight remained constant (dry weight). Samples were digested thoroughly with HNO_3_ at 180°C and the Cd^2+^ concentration was determined with an ICP Mass Spectrometer (NexION 350X, PerkinElmer).

### Determination of V-ATPase and V-PPase Activities

Root tissues (0.5 g) of four-week-old plants were used for V-ATPase and V-PPase activities determination according to Krebs et al. (2010) with some modifications. V-ATPase and V-PPase activities in 100 μl microsomal membranes were determined calorimetrically by measuring the release of inorganic phosphate (Pi) after an incubation of 30 min at 37°C. The V-ATPase assay medium contained 25 mM Tris-Hepes (pH 7.6), 3 mM MgSO_4_⋅7 H_2_O, 50 mM KCl, 0.5 mM NaN_3_, 0.1 mM NaVO_4_⋅12 H_2_O, and 3 mM ATP-Tris. V-PPase activity was assayed in a reaction medium containing 25 mM Tris-Hepes (pH 7.6), 3 mM MgSO_4_⋅7 H_2_O, 50 mM KCl, 0.5 mM NaN_3_, 0.1 mM NaVO_4_⋅12 H_2_O, and 3 mM Na_4_P_2_O_7_.

### Isolation of Intact Protoplasts and Vacuoles for Determination of Cd^2+^ and NO_3_^-^ Levels

Root tissues (0.3 g) of four-week-old plants were used to isolate intact protoplasts and vacuoles according to Robert et al. (2007). The purified protoplasts were divided into two fractions, one of which was used for the releases of vacuoles according to the method described in Dürr et al. (1975). The purified protoplasts and vacuoles were used for the determination of NO_3_^-^ and Cd^2+^ concentrations (Vögeli-Lange and Wagner, 1990; Han et al., 2016). NO_3_^-^ concentrations in protoplasts and vacuoles were measured by a continuous-flow auto-analyzer (Auto Analyzer 3, Bran and Luebbe) as described previously by Han et al. (2016). Cd^2+^ concentrations in the protoplasts and vacuole were determined with an ICP Mass Spectrometer (NexION 350X, PerkinElmer) as described in Huang et al. (2012).

### RNA Extraction and Transcript Analysis

Four-week-old plants were treated with 200 μM CdCl_2_ for 6 h and the roots were harvested for total RNA analysis. Total RNA was extracted with TRIzol (Invitrogen, United States), precipitated with an equal volume of isopropanol, washed with 75% ethanol, and dissolved with RNase-free water. The cDNA templates were synthesized using the PrimeScript^TM^ RT Kit with gDNA Eraser (Perfect Real Time) (TAKARA, Japan) following the manufacturer’s protocol. The relative expression of genes in roots was determined by quantitative RT-PCR performed in an Applied Biosystems StepOne^TM^ Real-Time PCR System using SYBR Premix Ex-Taq (TAKARA) according to the manufacturer’s protocol. Primers used in the assays are listed in Supplementary Table S1. The expression data were normalized to *Actin2* or *sand*.

### Statistical Analysis

A completely randomized design was applied in the experiments by having four biological replicates in each treatment. The comparisons of the means between Cd^2+^ stress treatments and controls were performed using the two-tailed Student’s *t*-test. The effects of Cd^2+^ stress treatment, along with the genotypes and their interactions, were evaluated using two-way analysis of variance (ANOVA). Multiple comparisons were performed using the least significant difference (*LSD*) multiple range test. Differences were considered statistically significant at *P* < 0.05.

## Results

### Nrt1.1 Improves Cd^2+^ Stress Tolerance in Plants

**FIGURE 1:**
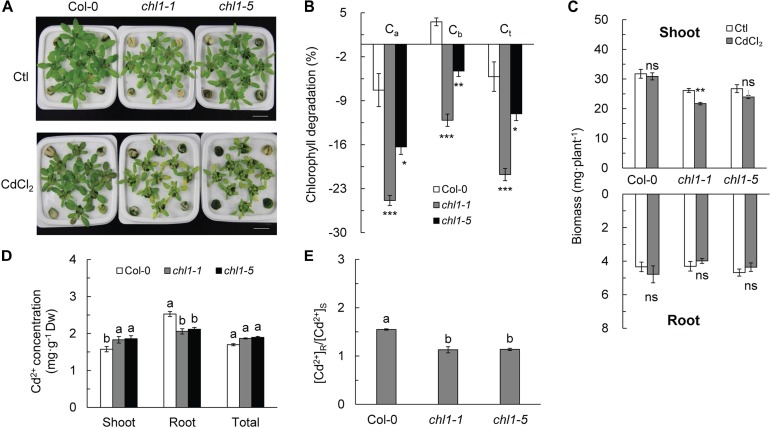
Effects of Cd^2+^ stress on growth and Cd^2+^ distribution in Col-0 and *nrt1.1* plants. **(A)** Images of four-week-old Col-0 and the *nrt1.1* plants, which were maintained in the absence (Ctl, control) or presence of 20 μM CdCl_2_ for 3 days. **(B)** Chlorophyll degradation rate (%) in CdCl_2_ treated plants relative to untreated control plants. **(C)** Shoot and root biomasses in Col-0 and *nrt1.1*. **(D)** Cd^2+^ concentration in Col-0 and *nrt1.1* plants maintained without or with 20 μM CdCl_2_ for 3 days. **(E)** [Cd^2+^] R:S ratio in Col-0 and *nrt1.1* plants. Data represent means ± SE (*n* = 4). Columns with the same letter indicate no significant difference at *P* < 0.05 using the *LSD* method. Scale bars = 1 cm. Bars with one (^∗^), two (^∗∗^), and three (^∗∗∗^) asterisks indicate significant differences from the control at *P* < 0.05, *P* < 0.01, and *P* < 0.001, respectively, using the two-tailed Student’s *t*-test.

Cd^2+^ stress degraded chlorophyll *a, b*, and total chlorophyll in *nrt1.1* mutants (*chl1-1* and *chl1-5*) by 16.35–24.87, 4.26–12.10, and 11.07–20.75%, respectively, as compared with those in the controls, resulting in more severe chlorosis in *nrt1.1* than in Col-0 (Figure 1A,B and Supplementary Figures S1A–C). Cd^2+^ stress significantly reduced shoot biomass of *nrt1.1*, but had no effect on Col-0; Cd^2+^ exposure did not affect the root biomass in any genotype (Figure 1C). *P*_n_, *V*_c,max_, *J*_max_, and TPU in *nrt1.1* were strongly decreased under Cd^2+^ stress, with significantly lower values than those in Col-0. Photosynthesis in Col-0 was not affected by Cd^2+^ stress (Supplementary Figures S2A–D). The *chl1-9* mutant is defective in nitrate uptake but shows a normal primary nitrate response (Ho et al., 2009). In the presence of Cd^2+^, *chl1-9* had chlorosis similar to *chl1-1* and *chl1-5* (Supplementary Figure S3), indicating that the nitrate signaling function of *NRT1.1* is independent of the underlying mechanism of Cd^2+^ stress tolerance in Arabidopsis.

Cd^2+^ stress strongly increased the MDA concentration in *nrt1.1*, especially in the roots, where it increased by 310.4% in *chl1-1* and 481.4% in *chl1-5* as compared to that in the corresponding controls. However, the MDA concentration in Col-0 was not affected in the shoots and only increased by 41.8% in the roots (Supplementary Figure S4A). Cd^2+^ stress significantly increased the proline concentration in all genotypes, with increases of 84.5 and 59.8%, 54.7 and 55.1%, and 35.4 and 43.3% observed in the shoots and roots of Col-0, *chl1-1*, and *chl1-5*, respectively, as compared to the proline concentration in the corresponding controls (Supplementary Figure S4B). Cd^2+^ stress significantly increased the SOD activity in the shoots of Col-0 but reduced it in the *nrt1.1* mutant, which had a significantly lower SOD activity than Col-0 (Supplementary Figure S4C).

We compared the concentration and distribution of Cd^2+^ in plants exposed to Cd^2+^ stress and found that the Cd^2+^ concentration was significantly lower in the shoots of Col-0 plants than in those of the *nrt1.1* plants. In contrast, the Cd^2+^ concentration in the roots of Col-0 plants was markedly higher than that of the *nrt1.1* plants (Figure 1D). However, the whole-plant Cd^2+^ concentration did not vary significantly between Col-0 and *nrt1.1* (Figure 1D). Therefore, the [Cd^2+^] R:S ratio was significantly higher in Col-0 than in *nrt1.1* (Figure 1E). These results indicated that Col-0 plants allocated more Cd^2+^ to the roots during Cd^2+^ stress, whereas *nrt1.1* plants distributed more Cd^2+^ to shoots, which was consistent with the observed Cd^2+^ toxicity in the shoots.

Since the iron (Fe) status of plants is critical for Cd^2+^ uptake and tolerance (He et al., 2017), we measured the Fe concentration but did not detect any genotype-dependent differences, neither under control nor Cd^2+^ stress conditions (Supplementary Figure S5). This observation suggested that the chlorosis in *nrt1.1* leaves under Cd^2+^ stress did not affect the Fe status.

### NRT1.1 Mediates Nitrate Allocation in Roots Under Cd^2+^ Stress

NRT1.1 is a nitrate transporter. To determine whether the phenotype of Cd^2+^ stress-induced chlorosis is nitrate-dependent, we used ammonium succinate as the sole nitrogen source during Cd^2+^ treatment and found that the phenotype of Cd^2+^ toxicity disappeared (Supplementary Figure S6). The results suggested that NRT1.1-mediated Cd^2+^ tolerance is nitrate-dependent.

**FIGURE 2:**
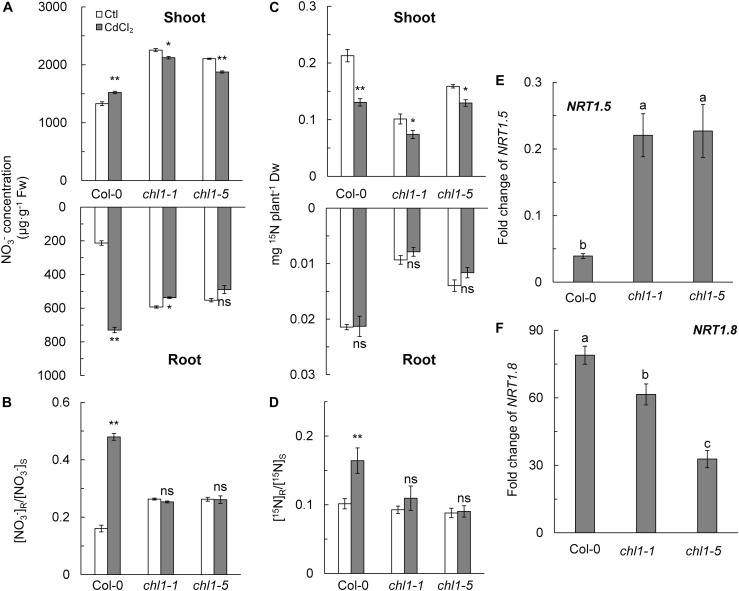
Effects of Cd^2+^ stress on nitrate allocation and the fold change of *NRT1.5* and *NRT1.8*. **(A)** NO_3_^-^ concentration in the shoots and roots of Col-0 and *nrt1.1*. **(B)** [NO_3_^-^] R:S ratio. **(C)** ^15^N levels in the shoots and roots. Four-week-old plants maintained without or with 200 μM CdCl_2_ for 12 h were labeled with 20% atom abundance of Ca(^15^NO_3_)_2_ for 40 min. **(D)** [^15^N] R:S ratio. **(E)** Fold change of *NRT1.5* expression calculated by dividing the expression of *NRT1.5* under CdCl_2_ treatment with the expression of *NRT1.5* in the control. **(F)** Fold change of *NRT1.8* expression calculated by dividing the expression of *NRT1.8* under CdCl_2_ treatment with the expression of *NRT1.5* in untreated controls. For the gene expression assay, plants were maintained without or with 200 μM CdCl_2_ for 6 h; roots were harvested for mRNA isolation. Data represent means ± SE (*n* = 4). Columns with the same letter indicate no significant difference at *P* < 0.05 using the LSD method. Scale bars = 1 cm. Bars with one (^∗^) and two (^∗∗^) asterisks indicate significant differences from the control at *P* < 0.05, and *P* < 0.01, respectively, using the two-tailed Student’s *t*-test.

A previous study reported that nitrate allocation in plants is correlated with Cd^2+^ stress tolerance (Li et al., 2010). Thus, we measured the NO_3_^-^ concentration and allocation in roots and shoots. The results showed that Cd^2+^ stress significantly reduced the NO_3_^-^ concentration in roots and shoots of *nrt1.1*, whereas the NO_3_^-^ concentration in roots and shoots of Col-0 was significantly increased by 2.43-fold and 0.13-fold as compared with those in control plants, respectively (Figure 2A). As a result, the [NO_3_^-^] R:S ratio in Col-0 was significantly increased by Cd^2+^ treatment, whereas this ratio remained constant in *nrt1.1* at approximately 0.26 (Figure 2B). The short-term ^15^N trace experiment further confirmed that allocation of absorbed nitrate to the roots was higher in Col-0 (14.00%) than in *chl1-1* (9.66%) or *chl1-5* (8.25%) under Cd^2+^ stress (Figure 2C,D).

The expression of nitrate long-distance transport genes *NRT1.5* and *NRT1.8* were also assayed in this study. As shown in Figure 2E and Supplementary Figure S7A, *NRT1.5* expression was strongly inhibited in Col-0 roots during Cd^2+^ exposure, whereas its expression was only slightly downregulated in *nrt1.1* mutants exposed to the same stress. In contrast, Cd^2+^ stress strongly upregulated the expression of *NRT1.8* in roots of all genotypes, with the highest expression levels measured in Col-0 (Figure 2F and Supplementary Figure S7B). The expression of *NRT1.5* and *NRT1.8* resulted in a higher [NO_3_^-^] R:S ratio under Cd^2+^ stress.

### *NRG2* Participates in *NRT1.1*-Mediated Nitrate Allocation

**FIGURE 3:**
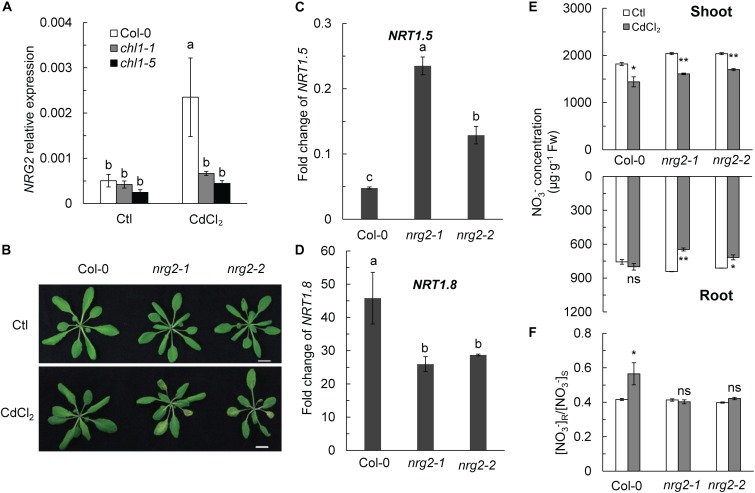
Phenotypes, NO_3_^-^ concentration, [NO_3_^-^] R:S ratio, and gene expression change in Col-0 and *nrg2*. **(A)** *NRG2* relative expression in Col-0 and *nrt1.1* in the absence or presence of Cd^2+^. **(B)** Images of four-week-old Col-0 and *nrg2* plants grown without or with 20 μM CdCl_2_ for 3 days. **(C)** Fold change of *NRT1.5* expression calculated as the expression of *NRT1.5* under CdCl_2_ treatment divided by the expression of *NRT1.5* in the untreated control. **(D)** Fold change of *NRT1.8* expression calculated as the expression of *NRT1.8* under CdCl_2_ treatment divided by the expression of *NRT1.5* in the untreated control. For the gene expression assay, plants were maintained without or with 200 μM CdCl_2_ for 6 h, roots were harvested for mRNA determination. **(E)** NO_3_^-^ concentration in shoot and roots of Col-0 and *nrg2* mutants. **(F)** [NO_3_^-^] R:S ratio. Data represent means ± SE (*n* = 4). Columns with the same letter indicate no significant difference at *P* < 0.05 using the LSD method. Scale bars = 1 cm. Bars with one (^∗^) and two (^∗∗^) asterisks indicate significant differences from the control at *P* < 0.05, and *P* < 0.01 respectively, using the two-tailed Student’s *t*-test.

Surprisingly, *NRG2* expression in Col-0 roots was also significantly upregulated by Cd^2+^ stress, whereas the expression of *NRG2* in *chl1-1* and *chl1-5* was not significantly affected (Figure 3A). Therefore, we hypothesized that *NRG2* is involved in *NRT1.1*-mediated Cd^2+^ tolerance in Arabidopsis. Interestingly, we observed the Cd^2+^ toxicity phenotype in two *NRG2* single mutants, *nrg2-1* and *nrg2-2*, after a 3-day treatment with 20 μM CdCl_2_ (Figure 3B).

The expression profile of *NRT1.5* and *NRT1.8* in *nrg2* mutants was similar to that in *chl1-1* and *chl1-5*. *NRT1.5* was more significantly downregulated in Col-0 than in *nrg2*, and *NRT1.8* was also more markedly upregulated in Col-0 than in the mutant (Figure 3C,D and Supplementary Figures S7C,D). Accordingly, Col-0 allocated more NO_3_^-^ to the roots and had an increased [NO_3_^-^] R:S ratio under Cd^2+^ stress (Figure 3E,F). In contrast, the NO_3_^-^ concentration was lower in *ngr2* shoots and roots than in control plant shoots and roots; thus, the *ngr2* mutant maintained a constant [NO_3_^-^] R:S ratio during the Cd^2+^ exposure (Figure 3E,F). These results indicated that the Cd^2+^ stress response in plants with depleted *NRG2* gene was similar to that in the *nrt1.1* mutant.

**FIGURE 4:**
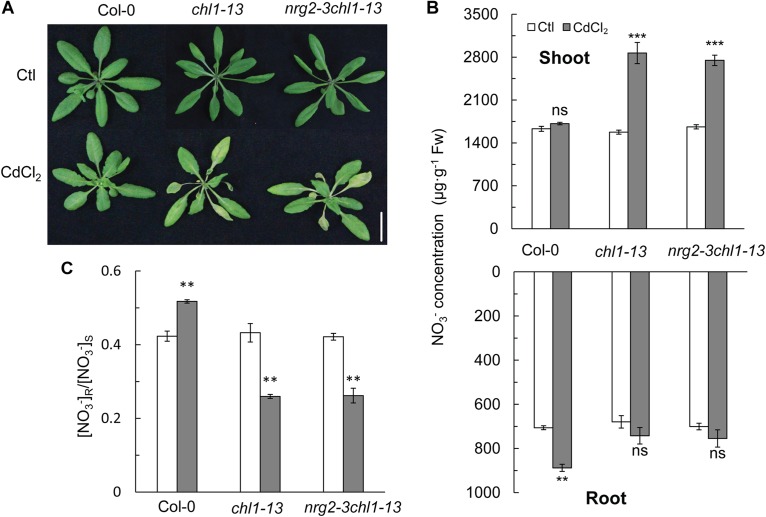
Phenotypes, NO_3_^-^ concentration, and [NO_3_^-^] R:S ratio in Col-0, *chl1-13*, and *nrg2chl1-13* mutants. **(A)** Images of phenotypes of Col-0, *chl1-13*, and *nrg2-3chl1-13* grown without or with 20 μM CdCl_2_ for 3 days. **(B)** NO_3_^-^concentration in Col-0, *chl1-13*, and *nrg2chl1-13* in shoots and roots. **(C)** [NO_3_^-^] R:S ratio of Col-0, *chl1-13*, and *nrg2chl1-13*. Data represent means ± SE (*n* = 4). Scale bars = 1 cm. Bars with two (^∗∗^) and three (^∗∗∗^) asterisks indicate significant differences from the control at *P* < 0.01, and *P* < 0.001, respectively, using the two-tailed Student’s *t*-test.

An *nrg2-3chl1-13* double mutant line was also used in this study. By exposing the double mutant plants to 20 μM Cd^2+^ for 3 days, we observed a Cd^2+^ toxicity phenotype that was similar to that of the *chl1-13* single mutant (Figure 4A). In the presence of Cd^2+^, the *nrg2-3chl1-13* mutant had an increased NO_3_^-^ concentration in the shoots and a decreased NO_3_^-^ concentration in the roots, which lowered the [NO_3_^-^] R:S ratio under Cd^2+^ stress (Figure 4B,C). The results indicated that *NRG2* and *NRT1.1* are involved in the same pathway regulating the expression of *NRT1.5* and *NRT1.8*, and, consequently, controlling NO_3_^-^ allocation and Cd^2+^ stress tolerance.

**FIGURE 5:**
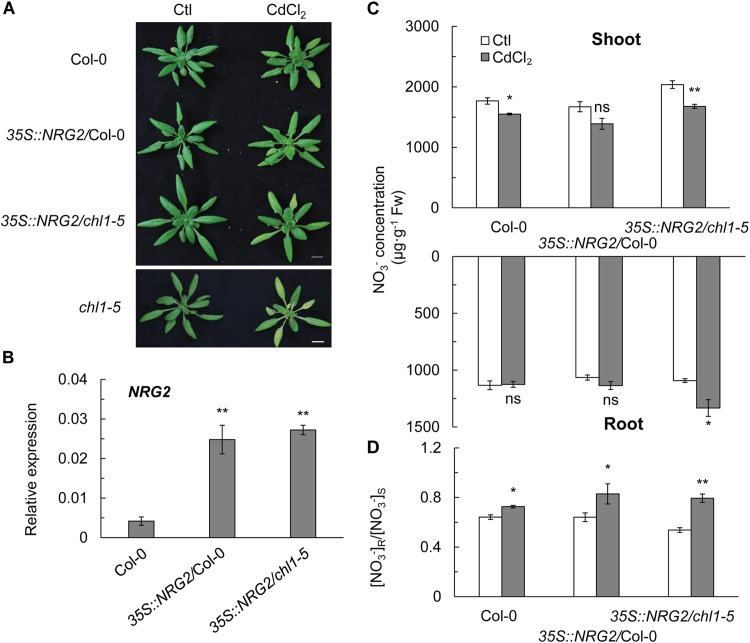
Phenotypes, NO_3_^-^ concentration, and [NO_3_^-^] R:S ratio in Col-0, *chl1-5*, and *NRG2* overexpression lines. Four-week-old plants were grown without or with 20 μM CdCl_2_ for 3 days, and shoots and roots were harvested for nitrate determination. **(A)** Phenotypes of Col-0, *35S::NRG2/*Col-0, *35S::NRG2/chl1-5*, and *chl1-5* in maintained in the absence or presence of 20 μM CdCl_2_. **(B)** *NRG2* relative expression in Col-0 and *NRG2* overexpression plants. **(C)** NO_3_^-^ concentration of Col-0, *35S::NRG2/*Col-*0*, and *35S::NRG2/chl1-5* in shoots and roots. **(D)** [NO_3_^-^] R:S ratio in Col-0 and *NRG2* overexpression plants. Scale bars = 1 cm. Data represent means ± SE (*n* = 4). Bars with one (^∗^) and two (^∗∗^) asterisks indicate significant differences from the control at *P* < 0.05, and *P* < 0.01, respectively, using the two-tailed Student’s *t*-test.

To further elucidate the relationship between *NRG2* and *NRT1.1* in regulating NO_3_^-^ allocation as a major response to Cd^2+^ stress, we constitutively overexpressed *NRG2* in Col-0 and *chl1-5* under the control of the 35S promoter. We found that Cd^2+^ stress did not induce the Cd^2+^ toxicity phenotype in *35S::NRG2*/Col-0 or *35S::NRG2*/*chl1-5*, both of which with an *NRG2* expression that was significantly higher than that in Col-0 (Figure 5A,B). Overexpression of *NRG2* in Col-0 and *chl1-5* reduced the NO_3_^-^ concentration in shoots but increased (or maintained) the NO_3_^-^ concentration in roots under Cd^2+^ stress (Figure 5C). Hence, the [NO_3_^-^] R:S ratio under Cd^2+^ stress in *35S::NRG2/*Col-0 and *35S::NRG2/chl1-5* was significantly increased as compared to that in Col-0 (Figure 5D). These data indicated that *NRG2* acted as a downstream element of *NRT1.1* to regulate NO_3_^-^ allocation under Cd^2+^ stress.

### Coordination of Cd^2+^ and NO_3_^-^ Allocation in Root Tissues

Cd^2+^ stress triggered both Cd^2+^ and NO_3_^-^ allocation in roots of Col-0 plants (Figure 1D,E, 2A,B, 3E,F). Previous studies showed that the cellular vacuoles function in nitrogen storage and heavy metal sequestration (Andreev, 2001; Sharma et al., 2016). In our study, we measured the distribution of Cd^2+^ and NO_3_^-^ in the vacuole and protoplast, along with the proton pump activity of the tonoplast, which promotes ion accumulation in the vacuole.

**FIGURE 6:**
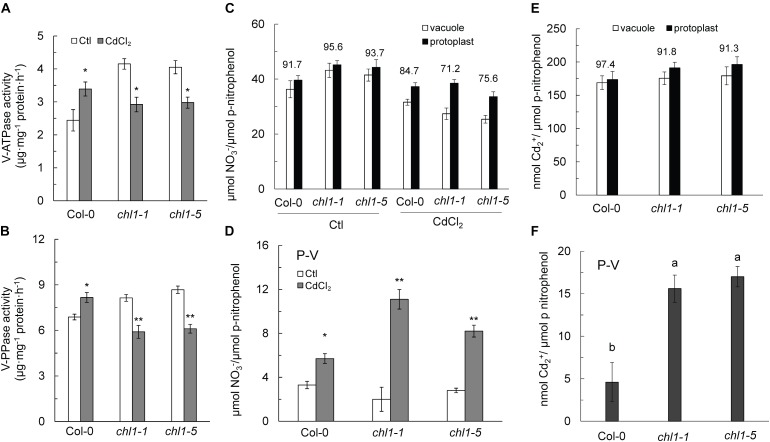
Reduced VSC of NO_3_^-^ and Cd^2+^ in the roots of *nrt1.1* mutant plants maintained without or with 20 μM CdCl_2_. **(A)** V-ATPase and **(B)** V-PPase (tonoplast proton-pump) activities in root tissue of Col-0 and *nrt1.1* mutant. **(C)** NO_3_^-^ distribution between the vacuole and protoplast in Col-0 and *nrt1.1* root tissue maintained without or with 20 μM CdCl_2_. **(D)** Total NO_3_^-^ in the cytosol under 20 μM CdCl_2_ treatment, calculated by total NO_3_^-^ in the protoplast minus total NO_3_^-^ in vacuoles. **(E)** Cd^2+^ distribution between the vacuole and protoplast in Col-0 and *nrt1.1* root tissue maintained without or with 20 μM CdCl_2_ treatment. **(F)** Total Cd^2+^ in the cytosol maintained without or with 20 μM CdCl_2_ treatment, calculated as the difference between total Cd^2+^ in the protoplast and total Cd^2+^ in vacuoles. Data represent means ± SE (*n* = 4). Values above the bars represent the percentage of total vacuolar NO_3_^-^ or Cd^2+^ in root tissue divided by total NO_3_^-^ or Cd^2+^ in total protoplasts in root tissue. Bars with one (^∗^) and two (^∗∗^) asterisks indicate significant differences from the control at *P* < 0.01, and *P* < 0.001, respectively, using the two-tailed Student’s *t*-test. Bars with the same letter indicate no significant difference at *P* < 0.05 using the *LSD* method.

As shown in Figure 6, the activities of the V-ATPase and V-PPase in roots tissues of Col-0 were increased under Cd^2+^ stress, whereas the activities of those two enzymes were significantly decreased in *chl1-1* and *chl1-5* roots (Figure 6A,B). Cd^2+^ stress reduced the NO_3_^-^ content in both protoplasts and vacuoles, with a greater reduction in the vacuole NO_3_^-^ content in *chl1-1* and *chl1-5* mutant plants, resulting in a lower proportion of vacuole NO_3_^-^ as compared to the total NO_3_^-^ in *nrt1.1* protoplasts (Figure 6C). NO_3_^-^ accumulation in the cytosol (P-V) was significantly increased in *nrt1.1* mutant plants and was markedly higher than that in Col-0 plants (Figure 6D).

Under Cd^2+^ stress, the proportion of vacuole Cd^2+^ relative to the total Cd^2+^ in root protoplasts was lower in *nrt1.1* than in Col-0 (Figure 6E), indicating that substantial amounts of Cd^2+^ accumulated in the cytosol of those *nrt1.1* root protoplasts (Figure 6F). Thus, we concluded that the increased allocation of NO_3_^-^ and Cd^2+^ to the root vacuoles is an effective strategy for Col-0 to increase the tolerance to Cd^2+^ stress.

## Discussion

### *NRT1.1* Improves Cd^2+^ Stress Tolerance by Nitrate Allocation in Roots

We found that Col-0 plants were more tolerant to Cd^2+^ stress than *nrt1-1* and *nrt1-5* plants. Interestingly, Col-0 also allocated more nitrate to roots, which was not observed in the *nrt1.1* mutant. It is well known that nitrate is reallocated to the roots under abiotic stress, including heavy metal stress (Hernandez et al., 1997). Nitrate reallocation to plant roots has been characterized as a typical mechanism that increases the stress tolerance in plants (Li et al., 2010; Chen et al., 2012). Our results suggested that the difference in nitrate allocation between Col-0 and *nrt1.1* is linked to their difference in Cd^2+^ tolerance. In this study, the data indicated that the enhanced nitrate allocation to the roots is correlated with transcript level changes of *NRT1.5* and *NRT1.8*, which corroborated earlier reports by Li et al. (2010) and Chen et al. (2012) using *nrt1.5* and *nrt1.8* mutants. Our results, along with the earlier reports, indicated that nitrate plays an essential role in the tolerance to Cd^2+^ stress via the regulatory function of NRT1.1.

Although nitrate allocation to roots improves stress tolerance in plants, the relevant physiological and molecular mechanisms are not well characterized. Cd^2+^ induces oxidative stress in plants (Mendoza-Cózatl et al., 2005; Hayat et al., 2007; Cuypers et al., 2010). Our results revealed that nitrate accumulation in roots is correlated with increases in antioxidant capacity, as indicated by less membrane lipid peroxidation, higher proline level, and stronger SOD activity (Supplementary Figures S2A–C), suggesting that the anti-oxidative system protected the plants from Cd^2+^ damage. As predicted, Col-0 maintained a higher photosynthetic rate (Supplementary Figure S1A). Nitrate reduction is an energy-intensive process (Sunil et al., 2013). Assimilation of nitrate in the leaves directly involves reducing compounds such as NADH, NADPH, reduced ferredoxin, and ATP derived from photosynthesis (Gonzalez-Dugo et al., 2010). Storage of nitrate in the roots can reduce the energy competition between nitrate reduction and photosynthesis, thus, reducing the adverse effect of Cd^2+^ on photosynthesis.

### NRG2 Works Together With NRT1.1 in the Regulation of Nitrate Allocation

*NRG2* is an important nitrate regulatory gene that has been reported to regulate nitrate signaling in Arabidopsis in part by modulating *NRT1.1* expression (Xu et al., 2016). The *nrg2* mutant showed lower nitrate accumulation in the roots than the wild-type due to reduced expression of *NRT1.1* and upregulation of *NRT1.8* (Xu et al., 2016). However, the relationship between these genes in regulating nitrate allocation remained unknown. Our results showed distinct expression patterns of *NRT1.5* and *NRT1.8* in Col-0 and the *nrt1.1* mutant, indicating that *NRT1.1* regulates the expression of *NRT1.5* and *NRT1.8*. Interestingly, we observed a significant upregulation of *NRG2* in Col-0 plants when grown under Cd^2+^ stress (Figure 3A), indicating that *NRG2* is involved in the response of nitrate-dependent Cd^2+^ stress.

By testing the *nrg2* single mutant, we observed a Cd^2+^ stress phenotype, and the expression patterns of *NRT1.5* and *NRT1.8* were similar to that in the *nrt1.1* mutant (Figure 3B–D). The *nrg2-3chl1-13* double mutant also had chlorosis similar to the *nrt1.1* plants and a reduced [NO_3_^-^] R:S ratio under Cd^2+^ stress (Figure 4A–C). The results indicated that *NRG2* and *NRT1.1* are involved in the same regulatory pathway for nitrate allocation under Cd^2+^ stress. Xu et al. (2016) reported that *NRG2* functions upstream of *NRT1.1* in nitrate signaling. By overexpressing the *NRG2* gene in *chl1-5* plants, we found that both Cd^2+^ stress induced chlorosis and nitrate allocation to the roots were restored in *35S::NRG2/chl1-5* plants (Figure 5A–D). The results demonstrated that *NRG2* functions downstream of *NRT1.1* to regulate Cd^2+^ stress-induced nitrate allocation. Our results also suggested that *NRG2* can cooperate with *NRT1.1* in nitrate signaling, depending on the growth conditions.

### Cd^2+^ Coordinates NO_3_^-^ Allocation to Root Vacuoles to Improve Cd^2+^ Stress Tolerance in Plant

Previous studies have demonstrated that stress-induced alterations of the expression of *NRT1.5* and *NRT1.8* depend on the presence of nitrate in the growth medium (Chen et al., 2012). Interestingly, the phenomenon of Cd^2+^ toxicity disappeared, as was also observed in previous studies, when the plants were supplied with ammonium as the sole nitrogen nutrient (Supplementary Figure S5). It indicated that Cd^2+^ toxicity is closely correlated with NO_3_^-^ allocation in plants. This observation was corroborated by studies performed in Arabidopsis, which showed that NO_3_^-^ and Cd^2+^ uptake increase simultaneously (Mao et al., 2014). Studies in rice showed that an excess of NO_3_^-^ increases the uptake and accumulation of Cd^2+^ (Yang et al., 2016). In this study, however, the uptake of Cd^2+^ in Col-0 was not different from that in *nrt1.1* mutants, but Col-0 plants allocated more Cd^2+^ to the roots (Figure 1D,E), which was consistent with the NO_3_^-^ allocation pattern (Figure 2A,B). These results suggest that the presence of NO_3_^-^ facilitates the allocation of Cd^2+^ to the roots, which increases the tolerance to Cd^2+^ stress.

The cellular vacuole plays important roles in maintaining the ion homeostasis in the plant cell and in regulating the responses to several abiotic and biotic stresses (Andreev, 2001). It acts as a storage pool of NO_3_^-^, in which the NO_3_^-^ concentration is an order of magnitude higher than that in the cytoplasm (Martinoia et al., 1981, 2000). Sequestration into the vacuole is one of the key mechanisms of heavy metal detoxification in plants (Sharma et al., 2016). The transfer of NO_3_^-^ and Cd^2+^ into the vacuole depends on a set of different transporters (De Angeli et al., 2006; Korenkov et al., 2006, 2007, 2009; Morel et al., 2009; Ueno et al., 2011). Nevertheless, both processes are mediated by V-ATPase and V-PPase energy pumps located on the membrane of tonoplast (Krebs et al., 2010; Sharma et al., 2016). We found that the activities of V-ATPase and V-PPase in Col-0 plants exposed to Cd^2+^ stress were significantly higher than those in the *nrt1.1* mutants (Figure 6A,B), leading to higher Cd^2+^ and NO_3_^-^ levels in root vacuoles (Figure 6C,E) and a stronger reduction of cytosolic Cd^2+^ and NO_3_^-^ levels in Col-0 plants as compared with those in *nrt1.1* plants (Figure 6D,F). Thus, there was less Cd^2+^ transport from the roots to the shoots, diminishing Cd^2+^ stress-induced injuries in the leaves. However, further studies are required to elucidate how the distribution of Cd^2+^ and NO_3_^-^ between root vacuoles and cytosol is regulated.

## Conclusion

Our results indicate that Cd^2+^ stress-initiated nitrate allocation to roots (SINAR) is associated with the antioxidant system to diminish stress-induced chlorosis. Furthermore, we found that root vacuoles are involved in the coordinated accumulation of Cd^2+^ and NO_3_^-^ under Cd^2+^ stress.

**FIGURE 7:**
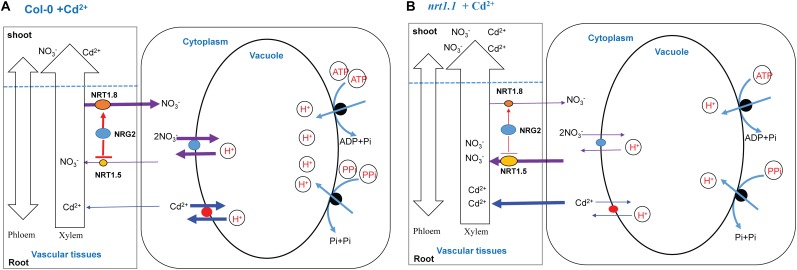
A schematic model of the coordinated regulation by *NRT1.1* and *NRG2* in plants exposed to Cd^2+^ stress. The red lines indicate the route of the regulation pathway. The size of arrows represents the flow rate. **(A)** When Col-0 plants are exposed to Cd^2+^, *NRT1.1* induces *NRG2* expression in roots and further downregulates *NRT1.5* expression and upregulates *NRT1.8* expression. As a result, more nitrate allocates to the roots. In addition, Cd^2+^ absorbed by the root system remains in the roots, and Cd^2+^ and NO_3_^-^ are coordinatively allocated between vacuole and cytosol by enhancing tonoplast proton-pump activities (V-ATPase and V-PPase). Thus, the Cd^2+^ and NO_3_^-^ contents are higher in the vacuole than in the cytoplasm, which facilitates Cd^2+^ detoxification and promotes growth. **(B)** In the *nrt1.1* mutant, *NRG2* expression is not changed by Cd^2+^ stress. The expression profiles of *NRT1.5* and *NRT1.8* are not affected as significantly as in Col-0 under Cd^2+^ stress. Hence, a greater proportion of nitrate absorbed by the root system is transported to the aboveground plant parts. Moreover, as compared with the control, the tonoplast proton-pump activities (V-ATPase and V-PPase) in *nrt1.1* mutants are lower under Cd^2+^ stress, resulting in enhanced Cd^2+^ and NO_3_^-^ allocation to the cytosol and transport to aboveground plant parts. Ultimately, *nrt1.1* plants are damaged under Cd^2+^ stress.

In Col-0 plants, *NRG2* acted as a downstream element of *NRT1.1* in the regulation of NO_3_^-^ allocation to the roots by downregulating *NRT1.5* and upregulating *NRT1.8* under Cd^2+^ stress. Moreover, a larger proportion of absorbed Cd^2+^ remained in the roots, where Cd^2+^ and NO_3_^-^ were allocated from the cytosol to the vacuole by increasing the tonoplast proton-pump activities (V-ATPase and V-PPase). Thus, intracellular sequestration reduced the transport of Cd^2+^ from roots to shoot, which is beneficial for Cd^2+^ detoxification and growth (Figure 7A).

In the *nrt1.1* mutant, *NRG2* expression was not changed by Cd^2+^ stress. Furthermore, the expression patterns of *NRT1.5* and *NRT1.8* in *nrt1.1* were not affected as significantly as those in Col-0 under Cd^2+^ stress. Hence, a larger proportion of NO_3_^-^ absorbed by the root system was transported to the aboveground parts. Moreover, as compared to those in the controls, the tonoplast proton-pump activities (V-ATPase and V-PPase) in the *nrt1.1* mutant were lower under Cd^2+^ stress, resulting in enhanced Cd^2+^ and NO_3_^-^ allocation to the cytosol and transport to aboveground parts. Ultimately, the *nrt1.1* mutant was more seriously injured under Cd^2+^ stress (Figure 7B). Our findings provide an insight into the underlying mechanism of the network of *NRT1.1* and *NRG2* in regulating Cd^2+^ stress tolerance in plants.

## Author Contributions

SJ and ZZ conceived the original screening and research plans and supervised the experiments. SJ performed most of the experiments and agreed to serve as the author responsible for contact and ensured communication. JL and QLiao provided technical assistance to SJ. SJ and ZZ designed the experiments and analyzed the data. QLiu and CG interpreted the result. ZZ conceived the project and wrote the article with contributions of all the authors and supervised and completed the writing.

## Conflict of Interest Statement

The authors declare that the research was conducted in the absence of any commercial or financial relationships that could be construed as a potential conflict of interest.

## Abbreviations

*J*_max_: maximum electron transport rate
MDA: malondialdehyde
NR: nitrate reductase
*NRG2*: *NITRATE REGULATORY GENE2*
PPED: photosynthetic photon flux intensity
R:S: root-to-shoot
SINAR: stress-initiated nitrate allocation in roots
SOD: superoxide dismutase
TPU: triose phosphate utilization
*V*_c,max_: maximum carboxylation rate of Rubisco

## References

[B1] Abouelsaad I. Weihrauch D. Renault S. (2016). Effects of salt stress on the expression of key genes related to nitrogen assimilation and transport in the roots of the cultivated tomato and its wild salt-tolerant relative. *Sci. Hortic.* 211 70–78. 10.1016/j.scienta.2016.08.005

[B2] Åkesson A. Barregard L. Bergdahl I. A. Nordberg G. F. Nordberg M. Skerfving S. (2014). Non-renal effects and the risk assessment of environmental cadmium exposure. *Environ. Health Persp.* 122 431–438. 10.1289/ehp.1307110 24569905 PMC4014752

[B3] Andreev M. (2001). Functions of the vacuole in higher plant cells. *Russ. J. Plant Physiol.* 48 777–787. 10.1023/a:1016776523371

[B4] Bates L. S. Waldren R. P. Teare I. D. (1973). Rapid determination of free proline for water-stress studies. *Plant Soil* 39 205–207. 10.1007/BF00018060 20688380

[B5] Bertin G. Averbeck D. (2006). Cadmium: cellular effects, modifications of biomolecules, modulation of DNA repair and genotoxic consequences. *Biochimie* 88 1549–1559. 10.1016/j.biochi.2006.10.001 17070979

[B6] Bouguyon E. Perrine-Walker F. Pervent M. Rochette J. Cuesta C. Benkova E. (2016). Nitrate controls root development through post-transcriptional regulation of the NRT1.1/NPF6.3 transporter/sensor. *Plant Physiol.* 172 1237–1248. 10.1104/pp.16.01047 27543115 PMC5047109

[B7] Cataldo D. A. Maroon M. Schrader L. E. Youngs V. L. (1975). Rapid colorimetric determination of nitrate in plant tissue by nitration of salicylic acid. *Commun. Soil Sci. Plant Anal.* 6 71–80. 10.1080/00103627509366547

[B8] Chaney R. L. (2015). How does contamination of rice soils with Cd and Zn cause high incidence of human Cd disease in subsistence rice farmers. *Curr. Pollut. Rep.* 1 13–22. 10.1007/s40726-015-0002-4

[B9] Chen C. Z. Lv X. F. Li J. Y. Yi H. Y. Gong J. M. (2012). *Arabidopsis* NRT1.5 is another essential component in the regulation of nitrate reallocation and stress tolerance. *Plant Physiol.* 159 1582–1590. 10.1104/pp.112.199257 22685171 PMC3425198

[B10] Chen H. M. (1996). *Heavy Metal Pollution in Soil–Plant System.* Beijing: Science Press.

[B11] Corratgé-Faillie C. Lacombe B. (2017). Substrate (un)specificity of *Arabidopsis* NRT1/PTR FAMILY (NPF) proteins. *J. Exp. Bot.* 68 3107–3113. 10.1093/jxb/erw499 28186545

[B12] Cuypers A. Plusquin M. Remans T. Jozefczak M. Keunen E. Gielen H. (2010). Cadmium stress: an oxidative challenge. *Biometals* 23 927–940. 10.1007/s10534-010-9329-x 20361350

[B13] De Angeli A. Monachello D. Ephritikhine G. Frachisse J. M. Thomine S. Gambale F. (2006). The nitrate/proton antiporter AtCLCa mediates nitrate accumulation in plant vacuoles. *Nature* 442 939–942. 10.1038/nature05013 16878138

[B14] Dürr M. Boller T. Wiemken A. (1975). Polybase induced lysis of yeast spheroplasts. A new gentle method for preparation of vacuoles. *Arch. Microbiol.* 105 319–327. 10.1007/BF00447152 242301

[B15] Fan X. Jia L. Li Y. Smith S. J. Miller A. J. Shen Q. (2007). Comparing nitrate storage and remobilization in two rice cultivars that differ in their nitrogen use efficiency. *J. Exp. Bot.* 58 1729–1740. 10.1093/jxb/erm033 17351248

[B16] Fang X. Z. Tian W. H. Liu X. X. Lin X. Y. Jin C. W. Zheng S. J. (2016). Alleviation of proton toxicity by nitrate uptake specifically depends on nitrate transporter 1.1 in *Arabidopsis*. *New Phytol.* 211 149–158. 10.1111/nph.13892 26864608

[B17] Giannopolitis C. N. Ries S. K. (1977). Superoxide dismutases I. occurrence in higher plants. *Plant Physiol.* 59 309–314. 10.2307/4264724 16659839 PMC542387

[B18] Gonzalez-Dugo V. Durand J. L. Gastal F. (2010). Water deficit and nitrogen nutrition of crops. A review. *Agron. Sustain. Dev.* 30 529–544. 10.1051/agro/2009059

[B19] Gowri G. Kenis J. D. Ingemarsson B. Redinbaugh M. G. Campbell W. H. (1992). Nitrate reductase transcript is expressed in the primary response of maize to environmental nitrate. *Plant Mol. Biol.* 18 55–64. 10.1007/bf00018456 1731978

[B20] Guo F. Q. Wang R. Chen M. Crawford N. M. (2001). The *Arabidopsis* dual-affinity nitrate transporter gene AtNRT1.1 (CHL1) is activated and functions in nascent organ development during vegetative and reproductive growth. *Plant Cell* 13 1761–1777. 10.2307/3871317 11487691 PMC139138

[B21] Guo F. Q. Wang R. Crawford N. M. (2002). The *Arabidopsis* dual-affinity nitrate transporter gene AtNRT1.1 (CHL1) is regulated by auxin in both shoots and roots. *J. Exp. Bot.* 53 835–844. 10.1093/jexbot/53.370.835 11912226

[B22] Guo F. Q. Young J. Crawford N. M. (2003). The nitrate transporter AtNRT1.1 (CHL1) functions in stomatal opening and contributes to drought susceptibility in *Arabidopsis*. *Plant Cell* 15 1–11. 10.1105/tpc.006312 12509525 PMC143464

[B23] Gutiérrez R. A. Gifford M. L. Poultney C. Wang R. Shasha D. E. Coruzzi G. M. (2007). Insights into the genomic nitrate response using genetics and the sungear software system. *J. Exp. Bot.* 58 2359–2367. 10.1093/jxb/erm079 17470441

[B24] Han Y. L. Song H. X. Liao Q. Yu Y. Jian S. F. Lepo J. E. (2016). Nitrogen use efficiency is mediated by vacuolar nitrate sequestration capacity in roots of *Brassica napus*. *Plant Physiol.* 170 1684–1698. 10.1104/pp.15.01377 26757990 PMC4775117

[B25] Hayat S. Ali B. Hasan S. A. Ahmad A. (2007). Brassinosteroid enhanced the level of antioxidants under cadmium stress in *Brassica juncea*. *Environ. Exp. Bot.* 60 33–41. 10.1016/j.envexpbot.2006.06.002

[B26] He Q. B. Singh B. R. (1994). Crop uptake of cadmium from phosphorus fertilizers: I. Yield and cadmium content. *Water Air Soil Pollut.* 74 251–265. 10.1007/BF00479793 27712871

[B27] He X. L. Fan S. K. Zhu J. Guan M. Y. Liu X. X. Zhang Y. S. (2017). Iron supply prevents Cd uptake in *Arabidopsis* by inhibiting IRT1 expression and favoring competition between Fe and Cd uptake. *Plant Soil* 416 453–462. 10.1007/s11104-017-3232-y

[B28] Hernandez L. E. Gárate A. Carpena-Ruiz R. (1997). Effects of cadmium on the uptake, distribution and assimilation of nitrate in *Pisum sativum*. *Plant Soil* 189 97–106. 10.1023/a:1004252816355

[B29] Hirel B. Le Gouis J. Ney B. Gallais A. (2007). The challenge of improving nitrogen use efficiency in crop plants: towards a more central role for genetic variability and quantitative genetics within integrated approaches. *J. Exp. Bot.* 58 2369–2387. 10.1093/jxb/erm097 17556767

[B30] Ho C. H. Lin S. H. Hu H. C. Tsay Y. F. (2009). CHL1 functions as a nitrate sensor in plants. *Cell* 138 1184–1194. 10.1016/j.cell.2009.07.004 19766570

[B31] Hu H. C. Wang Y. Y. Tsay Y. F. (2009). AtCIPK8, a CBL-interacting protein kinase, regulates the low-affinity phase of the primary nitrate response. *Plant J.* 57 264–278. 10.1111/j.1365-313X.2008.03685.x 18798873

[B32] Huang J. Zhang Y. Peng J. S. Zhong C. Yi H. Y. Ow D. W. (2012). Fission yeast HMT1 lowers seed cadmium through phytochelatin-dependent vacuolar sequestration in *Arabidopsis*. *Plant Physiol.* 158 1779–1788. 10.1104/pp.111.192872 22319073 PMC3320185

[B33] Korenkov V. Hirschi K. Crutchfield J. D. Wagner G. J. (2007). Enhancing tonoplast Cd/H antiport activity increases Cd, Zn, and Mn tolerance, and impacts root/shoot Cd partitioning in *Nicotiana tabacum* L. *Planta* 226 1379–1387. 10.2307/23389824 17636324

[B34] Korenkov V. King B. Hirschi K. Wagner G. J. (2009). Root-selective expression of AtCAX4 and AtCAX2 results in reduced lamina cadmium in field-grown *Nicotiana tabacum* L. *Plant Biotechnol. J.* 7 219–226. 10.1111/j.1467-7652.2008.00390.x 19175521

[B35] Korenkov V. Park S. Cheng N. H. Sreevidya C. Lachmansingh J. Morris J. (2006). Enhanced Cd2+-selective root-tonoplast-transport in tobaccos expressing *Arabidopsis* cation exchangers. *Planta* 225 403–411. 10.2307/23389558 16845524

[B36] Kramer G. F. Norman H. A. Krizek D. T. Mirecki R. M. (1991). Influence of UV-B radiation on polyamines, lipid peroxidation and membrane lipids in cucumber. *Phytochemistry* 30 2101–2108. 10.1016/0031-9422(91)83595-C

[B37] Krebs M. Beyhl D. Görlich E. Al-Rasheid K. A. Marten I. Stierhof Y. D. (2010). *Arabidopsis* V-ATPase activity at the tonoplast is required for efficient nutrient storage but not for sodium accumulation. *PNAS* 107 3251–3256. 10.1073/pnas.0913035107 20133698 PMC2840351

[B38] Krouk G. (2016). Hormones and nitrate: a two-way connection. *Plant Mol. Biol.* 91 599–606. 10.1007/s11103-016-0463-x 27003907

[B39] Krouk G. Crawford N. M. Coruzzi G. M. Tsay Y. F. (2010). Nitrate signaling: adaptation to fluctuating environments. *Curr. Opin. Plant Biol.* 13 265–272. 10.1016/j.pbi.2009.12.003 20093067

[B40] Li J. Y. Fu Y. L. Pike S. M. Bao J. Tian W. Zhang Y. (2010). The *Arabidopsis* nitrate transporter NRT1.8 functions in nitrate removal from the xylem sap and mediates cadmium tolerance. *Plant Cell* 22 1633–1646. 10.1105/tpc.110.075242 20501909 PMC2899866

[B41] Lin S. H. Kuo H. F. Canivenc G. Lin C. S. Lepetit M. Hsu P. K. (2008). Mutation of the *Arabidopsis* NRT1.5 nitrate transporter causes defective root-to-shoot nitrate transport. *Plant Cell* 20 2514–2528. 10.1105/tpc.108.060244 18780802 PMC2570733

[B42] Liu X. Cui H. Li A. Zhang M. Teng Y. (2015). The nitrate transporter NRT1.1 is involved in iron deficiency responses in *Arabidopsis*. *J. Plant Nutr. Soil Sci.* 178 601–608. 10.1002/jpln.201400480

[B43] Mao Q. Q. Guan M. Y. Lu K. X. Du S. T. Fan S. K. Ye Y. Q. (2014). Inhibition of nitrate transporter 1.1-controlled nitrate uptake reduces cadmium uptake in *Arabidopsis*. *Plant Physiol.* 166 934–944. 10.1104/pp.114.243766 25106820 PMC4213119

[B44] Martinoia E. Heck U. Wiemken A. (1981). Vacuoles as storage compartments of nitrate in barley leaves. *Nature* 289 292–294. 10.1038/289292a0

[B45] Martinoia E. Maeshima M. Neuhaus H. E. (2007). Vacuolar transporters and their essential role in plant metabolism. *J. Exp. Bot.* 58 83–102. 10.1093/jxb/erl183 17110589

[B46] Martinoia E. Massonneau A. Frangne N. (2000). Transport processes of solutes across the vacuolar membrane of higher plants. *Plant Cell Physiol.* 41 1175–1186. 10.1093/pcp/pcd059 11092901

[B47] Mendoza-Cózatl D. Loza-Tavera H. Hernández-Navarro A. Moreno-Sánchez R. (2005). Sulfur assimilation and glutathione metabolism under cadmium stress in yeast, protists and plants. *FEMS Microbiol. Rev.* 29 653–671. 10.1016/j.femsre.2004.09.004 16102596

[B48] Miller A. J. Fan X. Orsel M. Smith S. J. Wells D. M. (2007). Nitrate transport and signalling. *J. Exp. Bot.* 58 2297–2306. 10.1093/jxb/erm066 17519352

[B49] Mohammed A. S. Kapri A. Goel R. (2011). Heavy metal pollution: source, impact, and remedies. *Environ. Pollut.* 20 1–28. 10.1007/978-94-007-1914-9_1

[B50] Morel M. Crouzet J. Gravot A. Auroy P. Leonhardt N. Vavasseur A. (2009). AtHMA3, a P1B-ATPase allowing Cd/Zn/Co/Pb vacuolar storage in *Arabidopsis*. *Plant Physiol.* 149 894–904. 10.1104/pp.108.130294 19036834 PMC2633814

[B51] Mounier E. Pervent M. Ljung K. Gojon A. Nacry P. (2014). Auxin-mediated nitrate signalling by NRT1.1 participates in the adaptive response of *Arabidopsis* root architecture to the spatial heterogeneity of nitrate availability. *Plant Cell Environ.* 37 162–174. 10.1111/pce.12143 23731054

[B52] Ottosen L. M. Pedersen A. J. Hansen H. K. Ribeiro A. B. (2007). Screening the possibility for removing cadmium and other heavy metals from wastewater sludge and bio-ashes by an electrodialytic method. *Electrochim. Acta* 52 3420–3426. 10.1016/j.electacta.2006.06.048

[B53] Redinbaugh M. G. Campbell W. H. (1993). Glutamine synthetase and ferredoxin-dependent glutamate synthase expression in the maize (*Zea mays*) root primary response to nitrate (evidence for an organ-specifc response). *Plant Physiol.* 101 1249–1255. 10.1104/pp.101.4.1249 12231779 PMC160646

[B54] Remans T. Nacry P. Pervent M. Filleur S. Diatloff E. Mounier E. (2006). The *Arabidopsis* nrt1.1 transporter participates in the signaling pathway triggering root colonization of nitrate-rich patches. *PNAS* 103 19206–19211. 10.1073/pnas.0605275103 17148611 PMC1748200

[B55] Robert S. Zouhar J. Carter C. Raikhel N. (2007). Isolation of intact vacuoles from *Arabidopsis* rosette leaf-derived protoplasts. *Nat. Protoc.* 2 259–262. 10.1038/nprot.2007.26 17406583

[B56] Roberts T. L. (2014). Cadmium and phosphorous fertilizers: the issues and the science. *Procedia Eng.* 83 52–59. 10.1016/j.proeng.2014.09.012

[B57] Ruffel S. Gojon A. Lejay L. (2014). Signal interactions in the regulation of root nitrate uptake. *J. Exp. Bot.* 65 5509–5517. 10.1093/jxb/eru321 25165146

[B58] Sharkey T. D. Bernacchi C. J. Farquhar G. D. Singsaas E. L. (2007). Fitting photosynthetic carbon dioxide response curves for C3 leaves. *Plant Cell Environ.* 30 1035–1040. 10.1111/j.1365-3040.2007.01710.x 17661745

[B59] Sharma P. Dubey R. S. (2005). Modulation of nitrate reductase activity in rice seedlings under aluminium toxicity and water stress: role of osmolytes as enzyme protectant. *J. Plant Physiol.* 162 854–864. 10.1016/j.jplph.2004.09.011 16146311

[B60] Sharma S. S. Dietz K. J. Mimura T. (2016). Vacuolar compartmentalization as indispensable component of heavy metal detoxification in plants. *Plant Cell Environ.* 39 1112–1126. 10.1111/pce.12706 26729300

[B61] Sunil B. Talla S. K. Aswani V. Raghavendra A. S. (2013). Optimization of photosynthesis by multiple metabolic pathways involving interorganelle interactions: resource sharing and ROS maintenance as the bases. *Photosynth. Res.* 117 61–71. 10.1007/s11120-013-9889-z 23881384

[B62] Tsay Y. F. Schroeder J. I. Feldmann K. A. Crawford N. M. (1993). The herbicide sensitivity gene CHL1 of *Arabidopsis* encodes a nitrate-inducible nitrate transporter. *Cell* 72 705–713. 10.1016/0092-8674(93)90399-B 8453665

[B63] Ueno D. Milner M. J. Yamaji N. Yokosho K. Koyama E. Clemencia Z. M. (2011). Elevated expression of TcHMA3 plays a key role in the extreme Cd tolerance in a Cd hyperaccumulating ecotype of *Thlaspi caerulescens*. *Plant J.* 66 852–862. 10.1111/j.1365-313X.2011.04548.x 21457363

[B64] Undurraga S. F. Ibarrahenríquez C. Fredes I. Álvarez J. M. Gutiérrez R. A. (2017). Nitrate signaling and early responses in *Arabidopsis* roots. *J. Exp. Bot.* 68 2541–2551. 10.1093/jxb/erx041 28369507 PMC5854014

[B65] Vidal E. A. Moyano T. C. Canales J. Gutierrez R. A. (2014). Nitrogen control of developmental phase transitions in *Arabidopsis thaliana*. *J. Exp. Bot.* 65 5611–5618. 10.1093/jxb/eru326 25129132

[B66] Vögeli-Lange R. Wagner G. J. (1990). Subcellular localization of cadmium and cadmium-binding peptides in tobacco leaves: implication of a transport function for cadmium-binding peptides. *Plant Physiol.* 92 1086–1093. 10.2307/4272746 16667375 PMC1062420

[B67] Wang Y. Y. Hsu P. K. Tsay Y. F. (2012). Uptake, allocation and signaling of nitrate. *Trends Plant Sci.* 17 458–467. 10.1016/j.tplants.2012.04.006 22658680

[B68] Wellburn A. R. Lichtenthaler H. K. (1984). Formulae and program to determine total carotenoids and chlorophylls a and b of leaf extracts in different solvents. *Adv. Photosynth. Res.* 2 9–12. 10.1007/978-94-017-6368-4_3

[B69] Wong C. S. C. Li X. D. Zhang G. Qi S. H. Peng X. Z. (2003). Atmospheric deposition of heavy metals in the pearl river delta. *China. Atmos. Environ.* 37 767–776. 10.1016/s1352-2310(02)00929-9

[B70] Woodis T. C. Hunter G. B. Johnson F. J. (1977). Statistical studies of matrix effects on the determination of cadmium and lead in fertilizer materials and plant tissue by flameless atomic absorption spectrometry. *Anal. Chim. Acta* 90 127–136. 10.1016/S0003-2670(01)82301-1

[B71] Xu N. Wang R. Zhao L. Zhang C. Li Z. Lei Z. (2016). The *Arabidopsis* NRG2 protein mediates nitrate signaling and interacts with and regulates key nitrate regulators. *Plant Cell* 28 485–504. 10.1105/tpc.15.00567 26744214 PMC4790867

[B72] Yang Y. Xiong J. Chen R. Fu G. Chen T. Tao L. (2016). Excessive nitrate enhances cadmium (Cd) uptake by up-regulating the expression of OSIRT1, in rice (*Oryza sativa*). *Environ. Expl. Bot.* 122 141–149. 10.1016/j.envexpbot.2015.10.001

[B73] Zhang G. B. Yi H. Y. Gong J. M. (2014). The *Arabidopsis* ethylene/jasmonic acid-NRT signaling module coordinates nitrate reallocation and the trade-off between growth and environmental adaptation. *Plant Cell* 26 3984–3998. 10.1105/tpc.114.129296 25326291 PMC4247569

[B74] Zhang X. Y. Zhong T. Y. Liu L. Ouyang X. Y. (2015). Impact of soil heavy metal pollution on food safety in China. *PLoS One* 8:e0135182. 10.1371/journal.pone.0135182 26252956 PMC4529268

