# Supplementary material for: NRT1.1 Regulates Nitrate Allocation and Cadmium Tolerance in Arabidopsis
Source: Front Plant Sci. 2019 Mar 27;10:384. doi: 10.3389/fpls.2019.00384 (PMC6445965; doi:10.3389/fpls.2019.00384)
Supplement: Supplementary file 1 [file Data_Sheet_1.docx]

**Table S1.** List of primers used in this study.

| Genes | Primer | Sequence(5'-3') | Method |
| --- | --- | --- | --- |
| AT3G18780 | *ACTIN2F* | TGTGCCAATCTACGAGGGTTT | Real-time qPCR |
|  | *ACTIN2R* | TTTCCCGCTCTGCTGTTGT |  |
| AT2G28390 | *SANDF* | ATATGACACCCTTGCTTGGAGGGA |  |
|  | *SANDR* | TGAGAATAAGACACCAGACGCGCA |  |
| AT1G32450 | *NRT1.5F* | TGTCATTGGACTTTCATCGC |  |
|  | *NRT1.5R* | CCCACAACCTCTTGGTCTAATC |  |
| AT4G21680 | *NRT1.8F* | TCTTCATCTTCGCATACAGGCGGT |  |
|  | *NRT1.8R* | GCCATTATCGCAATCACAAGCCCA |  |
| AT3G60320 | *NRG2F* | AAGAACAGAATCAGCATCA |  |
|  | *NRG2R* | GACACCAACCATTGAGTA |  |


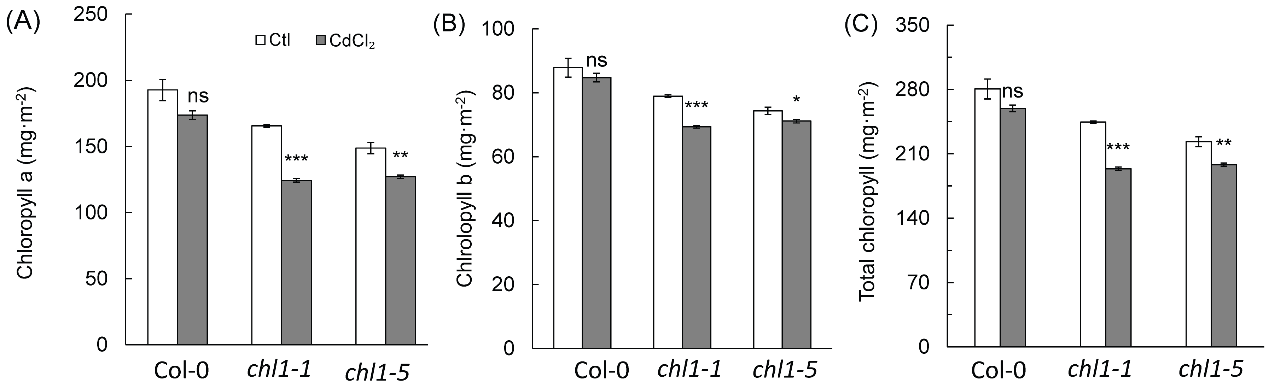


**Fig. S1** Effect of Cd^2+^ stress on chlorophyll a, b and total chlorophyll in Col-0 and *nrt1.1* mutants plants. **(A)** Chlorophyll a. **(B)** Chlorophyll b. **(C)** Total chlorophyll. Four-week-old Plants treated with and without 20 μM CdCl_2_ stress for 3 days and harvested for measurement. Data represent means ± SE (*n*=4). Bars with one (*), two (**) or three (***) asterisks indicate significant differences from the control at *P＜* 0.05, *P＜* 0.01, or *P＜* 0.001 respectively, according to Student’s t-test.


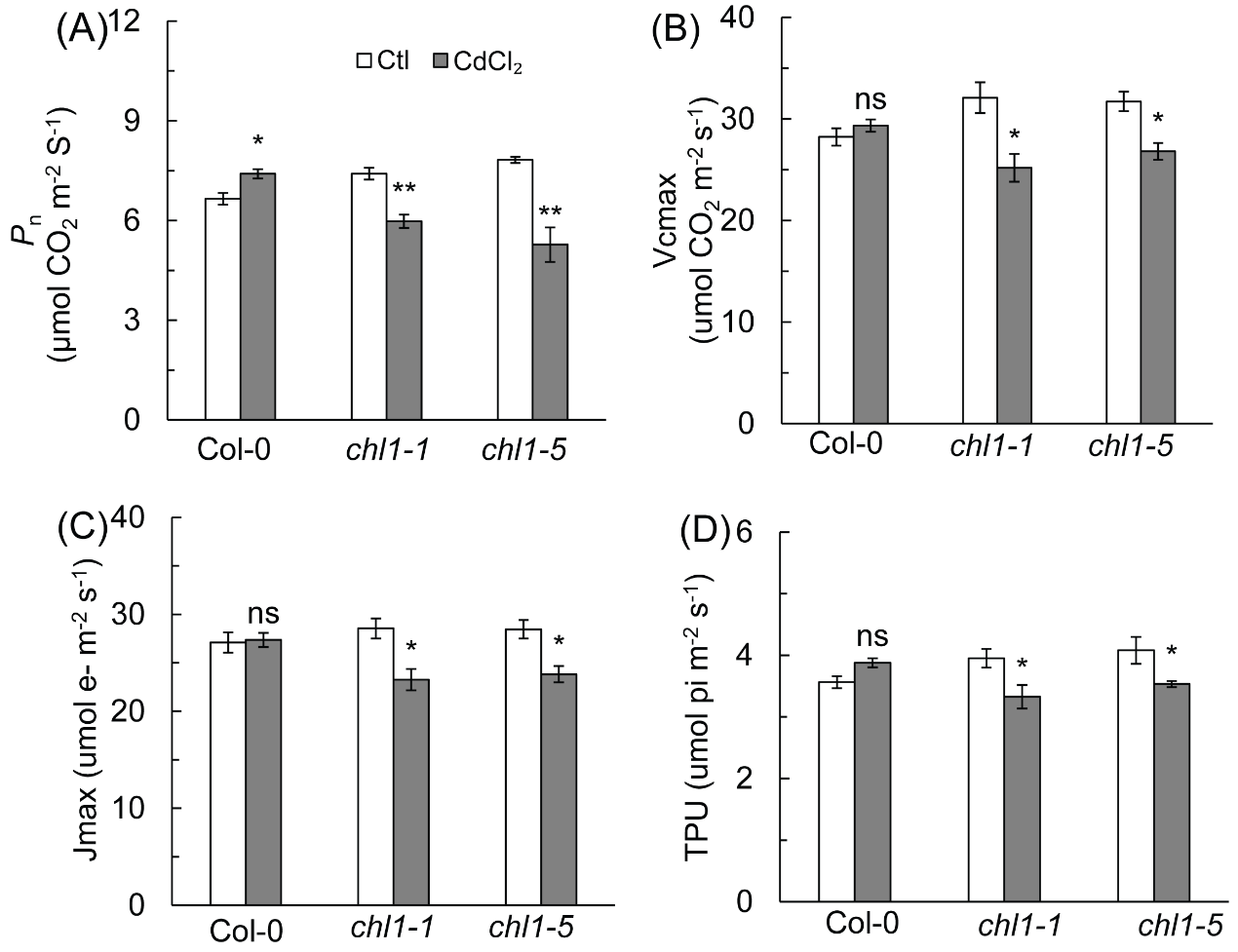


**Fig. S2** Effect of Cd^2+^ stress on photosynthesis in Col-0 and *nrt1.1* plants. (**A**) Net photosynthesis (**B**) Maximum carboxylation rate. (**C**) Maximum electron transport rate. (**D**) Triose utilization rate. Data represent means ± SE (*n*=4). Four-week-old Plants treated with and without 20 μM CdCl_2_ stress for 3 days and measured the photosynthesis system and CO_2_-response curves (*A-Ci curves*), calculated the Vcmax, Jmax, and TPU. Bars with one (*) or two (**) asterisks indicate significant differences from the control at *P＜* 0.05 or *P＜* 0.01, respectively, according to Student’s *t*-test.


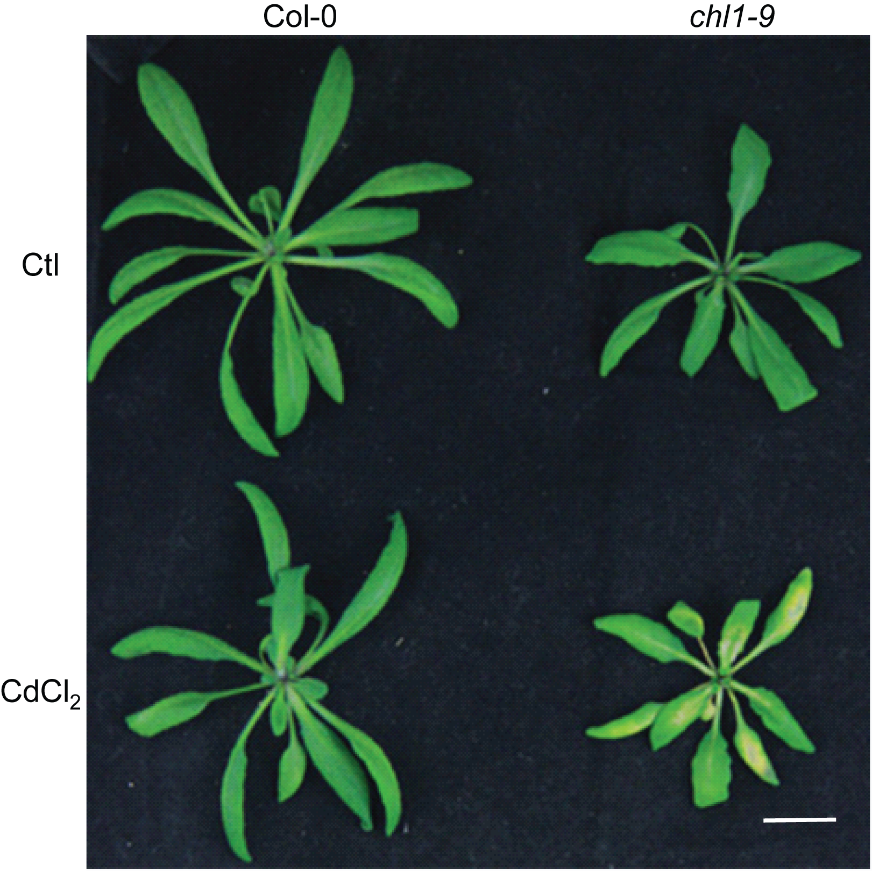


**Fig. S3.** Phenotypes of Col-0 and *chl1-9* treated with and without Cd^2+^ stress. Plants were grown as described in “Materials and methods” in the absence and presence of 20 μM CdCl_2_ for 3 days and pictured. Scale bar=1 cm.


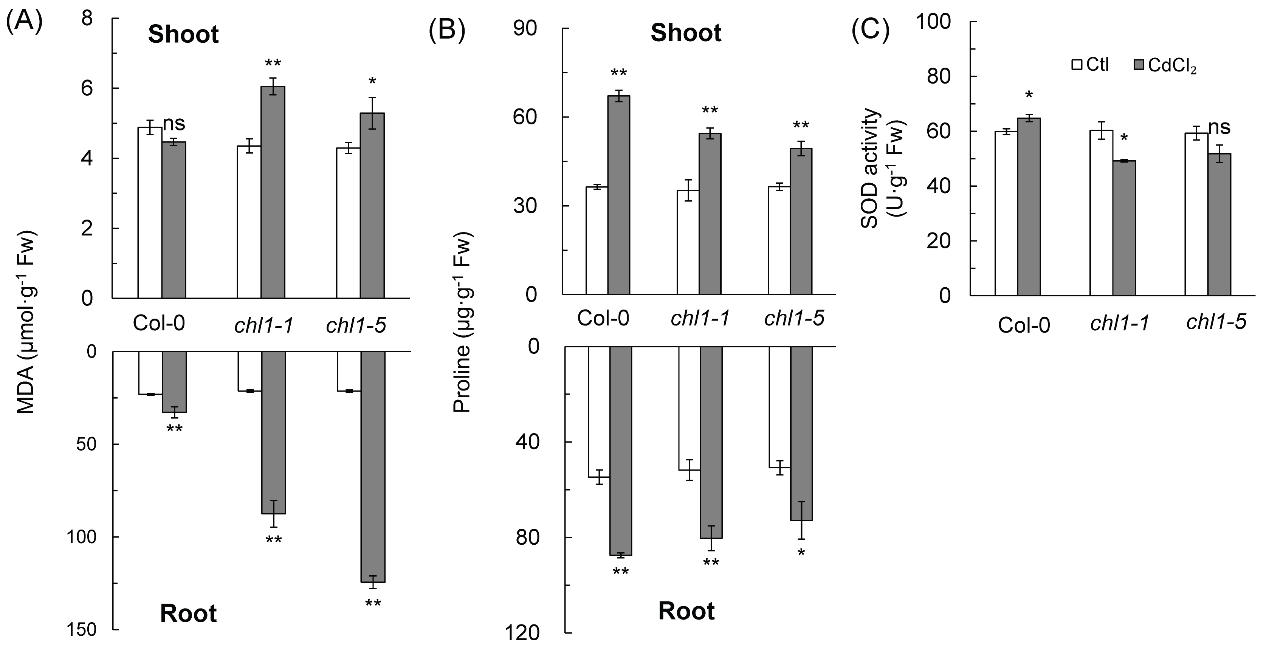


**Fig. S4.** Effect of Cd^2+^ stress on secondary metabolites in Col-0 and *nrt1.1* mutants plants. **(A)** Malondialdehyde (MDA). (**B**) Proline. (**C**) SOD activity. Four-week-old Plants treated with and without 20 μM CdCl_2_ stress for 3 days and harvested for measurement. Data represent means ± SE (*n*=4). Bars with one (*) or two (**) asterisks indicate significant differences from the control at *P＜* 0.05 or *P＜* 0.01, respectively, according to Student’s *t*-test.


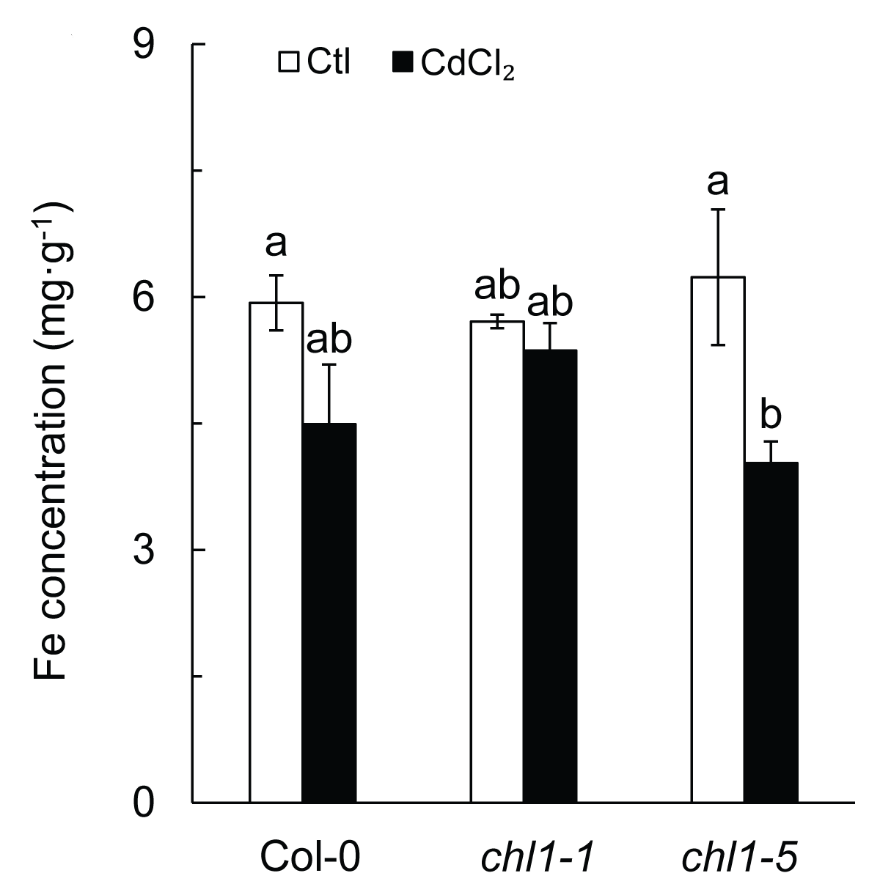


**Fig. S5.** Effect of Cd^2+^ stress on ferrum (Fe) concentration in Col-0 and *nrt1.1* mutants plants. Data represent means ± SE (*n*=4). Columns with the same letter indicate no significant difference at *P＜*0.05 using the *LSD* method.


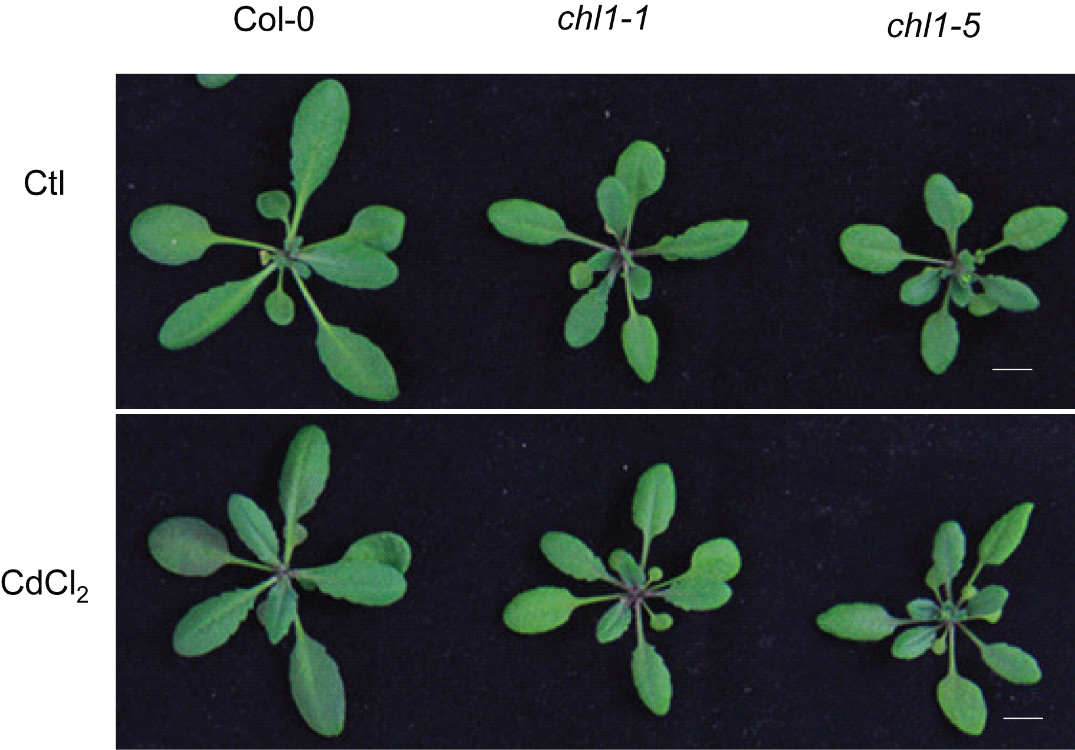


**Fig. S6.** Phenotypes of Col-0 and *nrt1.1* plants in ammonium succinate or ammonium succinate plus 20 μM CdCl_2_. Plants were grown as described in “Materials and methods” and kept under starvation for 24 h, after transfer to 2.25 mM ammonium succinate or ammonium succinate plus 20 μM CdCl_2_ for 3 days. Scale bar=1 cm.

**
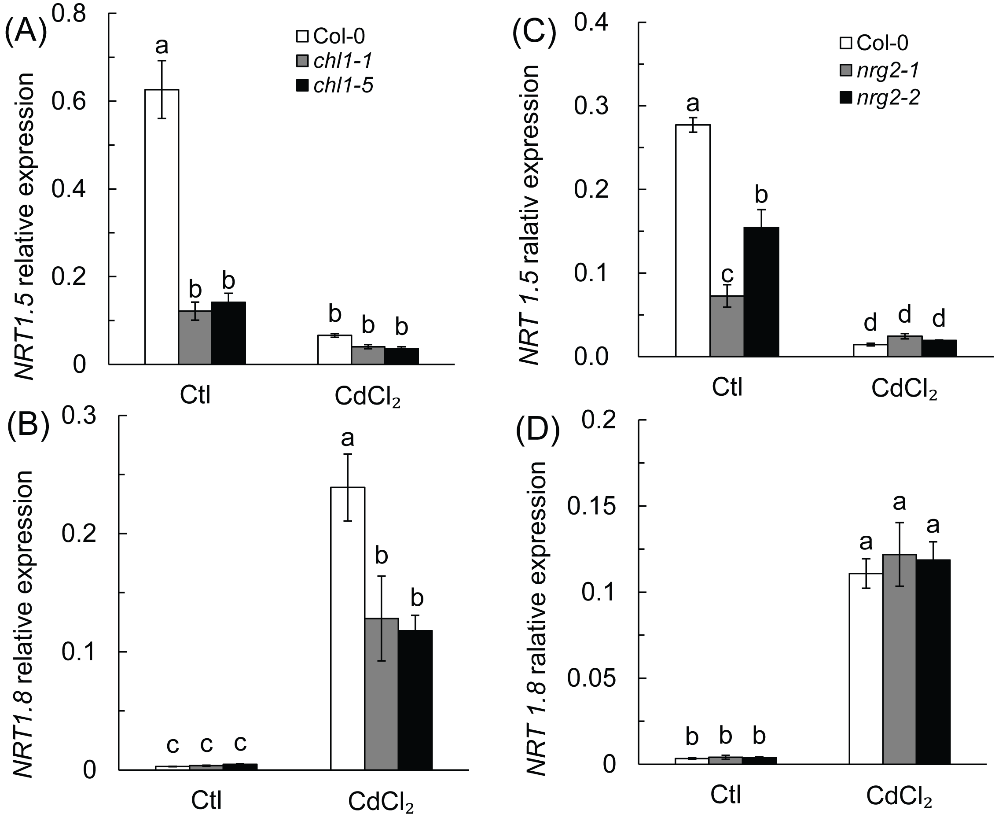
**

**Fig. S7.** Gene expression of *NRT1.5* and *NRT1.8* in roots of Co-0, *nrt1.1* mutants and *nrg2* mutants treated with and without Cd^2+^ stress. **(A)** and **(B)** Gene expression of *NRT1.5* and *NRT1.8* in roots of Col-0 and *nrt1.1* mutants. **(C)** and **(D)** Gene expression of *NRT1.5* and *NRT1.8* in roots of Col-0 and *nrg2* mutants. Four-week-old Plants treated with and without 200 μM CdCl_2_ for 6h and harvested for mRNA measurement. Data represent means ± SE (*n*=4). Bars with the same letter indicate no significant difference at *P＜* 0.05 using the *LSD* method.
